# Hemothorax following lung transplantation: incidence, risk factors, and effect on morbidity and mortality

**DOI:** 10.1186/s40248-016-0075-y

**Published:** 2016-11-15

**Authors:** Aria Hong, Christopher S. King, A. Whitney Walter Brown, Shahzad Ahmad, Oksana A. Shlobin, Sandeep Khandhar, Linda Bogar, Anthony Rongione, Steven D. Nathan

**Affiliations:** 1 Division of Pulmonary/Critical Care, University of California-Irvine, Irvine, USA; 2 Advanced Lung Disease and Transplant Clinic, Inova Fairfax Hospital, Falls Church, VA USA; 3 Cardiothoracic Surgery, Inova Fairfax Hospital, Falls Church, VA USA

**Keywords:** Hemothorax, Lung transplantation, Interstitial lung disease

## Abstract

**Background:**

Hemothorax after lung transplantation may result in increased post-operative morbidity and mortality. Risk factors for developing hemothorax and the outcomes of patients who develop hemothorax have not been well studied.

**Methods:**

A retrospective chart review was performed on all patients who underwent lung transplantation at a single center between March 2009 and July 2014. Comparison was made between patients with and without hemothorax post-transplant.

**Results:**

There were 132 lung transplantations performed during the study period. Hemothorax was a complication in 17 (12.9 %) patients, occurring an average of 9 days after transplant. No difference was found between the hemothorax and non-hemothorax groups with respect to age, preoperative anticoagulation, lung allocation score, prior thoracotomy, coagulation profile, use of cardiopulmonary bypass, ischemic time, or postoperative P/F ratio. There was a trend towards a higher incidence of hemothorax in patients with underlying sarcoidosis and re-transplantation (*p* = 0.13 and 0.17, respectively). Hemothorax developed early (<48 h post-operatively) in 5 patients and presented in a delayed manner (≥48 h post-operatively) in 12 patients. Delayed hemothorax occurred primarily in the setting of anticoagulation (10 out of 12 patients). The hemothorax group had decreased ventilator-free days (*p* = 0.006), increased ICU length of stay (*p* = 0.01) and increased hospital length of stay (*p* = 0.005). Hemothorax was also associated with reduced 90-day survival (*p* = 0.001), but similar 1, 3, and 5-year survival (*p* = 0.63, *p* = 0.30, and *p* = 0.25), respectively).

**Conclusion:**

The development of hemothorax is associated with increased morbidity and decreased short-term survival. Hemothorax may present either within the first 48 h after surgery or in a delayed fashion, most commonly in the setting of anticoagulation.

## Background

Lung transplantation may be an option for patients with various forms of advanced lung disease and is undertaken with the goal of improving both survival as well as quality of life [1]. Although the procedure holds the promise of improved outcomes, a number of complications may arise including primary graft dysfunction, infection, rejection, and venous thromboembolism. Post-transplant management often involves a delicate balance between the likelihood of various complications and the potential risks of prophylactic therapies themselves. The most common example of the “transplantation tightrope” is the titration of immunosuppressive medications to prevent rejection without exposing the transplant recipient to undue risk of infection. An equally common, but less discussed, balance which must be considered is the risk of bleeding versus thrombosis. Particular attention has been paid to the prevention of venous thromboembolism post-transplantation given a reported high incidence and the potential for adverse outcomes [2]. Patients are also at risk for post-operative bleeding following lung transplantation given the need for anticoagulation and potential for thrombocytopenia and coagulopathy associated with cardiopulmonary bypass, as well as the extensive surgical resection required for native lung extraction in many end-stage lung diseases [3].

The development of hemothorax appears to be common, complicating 17.8 % of transplants in a series of 107 patients with chronic obstructive pulmonary disease (COPD) [4]. The limited data available also suggest post-transplant hemothorax is associated with post-operative morbidity and mortality. In a retrospective study of 100 patients, hemothorax was shown to occur in 15 % of patients after lung transplantation, with 46 % of patients requiring surgery for massive hemothorax and an associated 40 % in hospital mortality [5]. In another series, 1 month mortality after bilateral lung transplantation for emphysema was found to be higher in patients who developed a hemothorax [6]. These results suggest that hemothorax is a serious complication that affects patient outcomes and requires special clinical attention. The patient characteristics that increase the likelihood of developing hemothorax have not been well studied. Additionally, the impact of hemothorax on long-term survival remains unknown. We aimed to identify patient characteristics associated with the development of post-transplant hemothorax and study the impact of this complication on patient morbidity and mortality.

## Methods

### Study design and data collection

An extensive chart review was conducted of all patients receiving a lung transplant at our tertiary care center between March 2009 and July 2014. We limited our definition of hemothorax to only those that were regarded as “significant”. We defined a significant hemothorax as one that required an intervention, including tube thoracostomy, surgery, vasopressors or transfusion. The diagnosis of hemothorax based on sanguineous chest tube output or radiographic findings alone were not included given some variability in reporting and the known expectation of some bleeding post-operatively. Data collection was performed by one investigator (AH). If the diagnosis of hemothorax was questionable, the case was reviewed by two attending pulmonologists (CK and SN) and a consensus was achieved. Transfusion and vasopressors were considered to be related to hemothorax if they were administered in the twelve hours before or after intervention or discovery of the hemothorax. In the event no procedural intervention was required, the period twelve hours prior to and after the clinical diagnosis of hemothorax was scrutinized for medical interventions deemed hemothorax-related. The outcomes studied focused on morbidity and mortality and included the following: intensive care unit (ICU) length of stay (LOS), hospital LOS, ventilator-free days, and 90-day, 1-year, 3-year, and 5-year survival.

Baseline demographics including preoperative, intraoperative, and postoperative characteristics of patients who developed hemothorax were compared to those who did not. Demographic and other patient-specific variables recorded included age, sex, lung allocation score (LAS), pre-transplant hemoglobin and platelet count, pre-operative use of prophylactic or therapeutic anticoagulation, pre-operative use of antiplatelet agents, history of prior thoracic surgery [excluding video-assisted thorascopic surgery (VATS) lung biopsy], underlying primary lung disease, single or bilateral transplant, initial transplant or re-transplantation, mean pulmonary artery pressure (mPAP), and pre-operative need for mechanical ventilation. Intra-operative and post-operative variables assessed included ischemic time, surgical description of adhesions (moderate or dense), need for cardiopulmonary bypass, need for and quantity of intra-operative packed red blood cell transfusion, post-operative international normalized ratio (INR), partial thromboplastin time (PTT), hemoglobin, and platelet count, and 72 h pulmonary arterial oxygen saturation to fraction of inspired oxygen ratio (P/F ratio). The post-operative lab data reported was obtained from testing performed immediately upon arrival to the ICU from the operating room. In addition, we compared the characteristics of “early” (defined as < 48 h post-operatively) and “delayed” (≥48 h post-transplant) hemothorax patients. Aspects of post-transplant morbidity assessed included ICU LOS, hospital LOS, chest tube duration, and ventilator-free days. This study was approved by the institutional review board (IRB) of our institution (IRB# 13.1394).

### Standard clinical practices

Initiation of prophylactic and therapeutic anticoagulation at our institution is individualized to patients after a risk/benefit analysis by the transplant team. In general, chemical deep vein thrombosis (DVT) prophylaxis at our institution in the form of subcutaneous heparin 5000 units three times daily is initiated 48–72 h post-operatively. When therapeutic anticoagulation is indicated, it is typically initiated in the form of an intravenous heparin infusion with a PTT goal of 60 to 90 s. The decision to use cardiopulmonary bypass is individualized and ultimately determined by the cardiac anesthesiologist and cardiothoracic surgeon performing the transplant. Traditional cardiopulmonary bypass, rather than extracorporeal membrane oxygenation (ECMO) support, was used for all patients requiring cardiopulmonary bypass at our institution at the time of data collection. Single lung-transplantation is performed through a posterolateral thoracotomy and bilateral transplant is performed through anterior thoracosternotomy (“clamshell incision”). Post-operative resuscitation and correction of coagulopathy/thrombocytopenia is performed at the discretion of the cardiothoracic/transplant surgery team after consideration of the degree of surgical bleeding.

### Statistical analysis

Descriptive statistics and bivariate analyses were performed using 2-sample t-tests for continuous patient characteristics and Fisher’s exact test for categorical patient characteristics. Survival time was calculated from the time of lung transplantation. Kaplan- Meier survival curves were generated and compared using the Log-Rank test. Overall 90-day survival was first examined. Next, 1-year survival (overall and contingent on 90-day survival) as well as 3 and 5-year survival were examined for lung transplant recipients. Univariate and multivariate analyses using Cox proportional hazard modeling were performed to further examine the relationship between hemothorax and survival adjusted for baseline patient characteristics. Survival analyses were performed using STATA version 12 (StataCorp LP; College Station, TX, USA). All tests were two-tailed and *p*-values of less than 0.05 were considered significant.

## Results

A total of 132 lung transplantations in 129 patients (3 re-transplantations) were included in the review (Table 1). Significant hemothorax developed in 17 of the 132 cases (12.9 %) and occurred from 0 to 32 days after transplant with a mean of 9.4 days and median of 8 days. Table 2 provides individualized data about the 17 patients with hemothorax. Veno-venous ECMO support was utilized pre-operatively for one patient and post-operatively for one patient with primary graft dysfunction.

**Table 1:** Patient characteristics of the hemothorax (HTX) and non-hemothorax (No HTX) group with corresponding *p* values

Characteristics	HTX (*N* = 17)	No HTX (*N* = 115)	*P*
Age (years)	52	55	0.39
Gender (male)	13/17 (76 %)	55/114 (48 %)	0.038
LAS	57	51	0.27
Previous thoracotomy	7/17 (41 %)	33/115 (29 %)	0.40
Bilateral Lung Transplant	6/17 (35 %)	33/115 (29 %)	0.58
Pre-tx mPAP	27	29	0.41
Pre-tx Hemoglobin	12.7	12.3	0.51
Pre-tx Platelet	242,000	259,000	0.52
Pre-tx Full Anticoagulation	1/17 (6 %)	9/115 (8 %)	1.00
Pre-tx Aspirin	4/17 (24 %)	26/115 (23 %)	1.00
Pre-tx Mechanical Ventilation	1/17 (6 %)	4/115 (3 %)	0.50
Ischemic Time (minutes)	221	186	0.15
Use of Cardiopulmonary Bypass	11/17 (65 %)	65/115 (57 %)	0.61
Moderate-dense adhesions	7/17 (41 %)	33/115 (29 %)	0.40
Intraoperative Transfusion: Units of PRBCs	2.0	0.9	0.24
Post-tx Hemoglobin	10.5	10.6	0.78
Post-tx Platelet	157,000	168,000	0.49
Post-tx INR	1.3	1.4 (*N* = 113)	0.21
Post-tx PTT	40	37	0.66
P/F Nadir by 72 hours	224	256	0.19
Underlying Lung Disease:			
COPD	1	19	NS
Bronchiectasis	0	4	
IPF	6	39	
ILD	3	32	
Sarcoidosis	4	12	
Re-transplantation	2	4	
Other	1	5	

*COPD* Chronic Obstructive Lung Disease, *ILD* Interstitial lung disease, *IPF* Idiopathic pulmonary fibrosis, *LAS* lung allocation score, *mPAP* mean pulmonary artery pressure, *NS* Not significant, *PRBCs* packed red blood cells, *P/F* PaO_2_/FiO_2_ ratio, *TX* transplant

**Table 2:** Details of patients with hemothorax

	Bleed date	AC date	AC	AC Dosing	PRBCs (units)	Intervention
1	0	-	-	-	6	Thoracotomy
2	0	-	-	-	2	Thoracotomy
3	1	-	-	-	5	Thoracotomy
4	1	-	-	-	3	Thoracotomy
5	1	-	-	-	5	VATS
6	4	-	-	-	2	none
7	6	1	Heparin		0	VATS
8	6	2	Heparin	Prophylactic	2	VATS
9	8	4	Heparin	Prophylactic to therapeutic	0	Chest tube
10	8	-	-	-	8	VATS
11	9	2	Heparin	Prophylactic	2	VATS
12	10	3	Heparin	Prophylactic	2	VATS
13	13	3	Heparin then warfarin	Prophylactic to therapeutic	8	Thoracotomy
14	15	3	Heparin	Prophylactic to therapeutic	3	Chest tube
15	15	0	LMWH	Prophylactic	0	Chest tube
16	31	4	Heparin to warfarin	Therapeutic	2	Thoracentesis
17	32	4	Heparin	Prophylactic to therapeutic	4	Died from hemothorax

*AC* Anticoagulation, *BLTx* Bilateral Lung Transplant, *LMWH* Low-molecular weight heparin, *SLTx* Single Lung Transplant, *VATS* Video-assisted thorascopic surgery

Of the 17 patients who developed a significant hemothorax, surgical re-exploration was deemed necessary in 11. The site of bleeding was identified in only three cases and included bleeding from a bronchial artery, an intercostal artery and chest wall bleeding at the site of the thoracotomy.

Of the remaining 6 patients, 3 required a new chest tube and one had a thoracentesis. Patients required an average of 3 units of packed red blood cell around the time of the hemothorax (up to 6 days before hemothorax and 2 days after hemothorax). The number of blood transfusions required for the hemothorax was determined by review of the individual chart. Transfusion requirements may be underestimated as some patients were noted to have decreasing hemoglobin and hematocrit and were transfused blood products for several days prior to the diagnosis of hemothorax.

The patients who developed hemothorax were divided into two groups – early hemothorax (within 48 h post-transplant, *N* = 5) and delayed hemothorax (≥48 h post-transplant, *N* = 12). Most patients who developed a late hemothorax were on anticoagulation prior to developing the hemothorax (10/12 or 83 %).

### Association of patient characteristics with development of hemothorax

#### Patient and pre-operative variables

Characteristics of patients who did and did not develop a hemothorax were compared (Table 1). The only statistically significant difference between the two groups was there were more males (76 % vs. 48 %, *p* = 0.038) in the group with hemothorax. There were no significant differences in patient age between groups. The average lung allocation score (LAS) was 57 in the hemothorax group and 51 in the non-hemothorax group (*p* = 0.27). Forty-one percent of patients (7 out of 17) had prior chest surgery in the hemothorax group, compared to 29 % (33 out of 115) in the non-hemothorax group (*p* = 0.40). Thirty-five percent (6 out of 17) in the hemothorax group had a bilateral lung transplant as compared with 29 % (33 out of 115) in the non-hemothorax group who had a bilateral lung transplant (*p* = 0.58). The mean pre-transplant pulmonary artery pressure (mPAP) was 27 mmHg in the hemothorax group and 29 mmHg in the non-hemothorax group (*p* = 0.41). There were no significant differences in pre-transplant hemoglobin (*p* = 0.51), platelet count (*p* = 0.52), or post-transplant hemoglobin (*p* = 0.78), platelet count (*p* = 0.49), or INR (*p* = 0.21). There was also no difference in pre-transplant anticoagulation, with 6 % (1 out of 17) of the hemothorax group and 8 % (9 out of 115) in the non-hemothorax group receiving therapeutic anticoagulation prior to transplant (*p* = 1.0). Likewise, treatment with aspirin was similar pre-transplantation (4/17 or 24 %) in the hemothorax and non-hemothorax groups (26/115 or 23 %) (*p* = 1.0). There was no difference in pre-transplant ventilator dependence, with 6 % (1/17) in the hemothorax group, and 3 % (4/115) in the non-hemothorax group on mechanical ventilation prior to transplant (*p* = 0.50).

#### Perioperative variables

Ischemic time was not different between the two groups, although there was a trend towards significance with the mean ischemic time of 221 min in the hemothorax group and 186 min in the non-hemothorax group (*p* =0.15). Sixty-five percent (11/17) of the hemothorax group required cardiopulmonary bypass compared to 57 % (65/115) in the non-hemothorax group (*p* = 0.61). Operative reports were also studied to assess the density of adhesions encountered during the lung transplant. If the operative reports did not mention adhesions, it was assumed that the amount of adhesions was minimal. Moderate to dense adhesions were present in 41 % (7/17) in the hemothorax group, and 29 % (33/115) in the non-hemothorax group (*p* = 0.4). In addition, the average number of intraoperative packed red blood cell transfusion was 2.0 in the hemothorax group compared to 0.9 in the non-hemothorax group (*p* = 0.24).

#### Postoperative variables

Postoperatively, the effect of primary graft dysfunction on the development of hemothorax was investigated by comparison of the nadir P/F ratio at 72 h between the two study groups. There was no difference in P/F ratio, which was 224 in the hemothorax group and 256 in the non-hemothorax group (*p* = 0.19). No statistically significant difference was found for postoperative hemoglobin, platelet count, INR, or PTT.

#### Underlying lung disease

The rate of hemothorax based on underlying lung disease was also evaluated. Hemothorax developed in 5 % of patients with COPD (1 out of 20), 0 % of patients with bronchiectasis (0 out of 4), 13 % of patients with idiopathic pulmonary fibrosis (IPF) (6 out of 45), 9 % of patients with other interstitial lung disease (ILD) (3 out of 35), 25 % of patients with sarcoidosis (4 out of 16), 33 % of re-transplants (2 out of 6), and 16 % of other lung disease (1 out of 6). The other lung disease group included one patient with bronchopulmonary dysplasia, one patient with alveolar proteinosis, 3 patients with lymphangioleiomyomatosis (LAM), and one patient with graft versus host disease after a bone marrow transplant, which is the patient who developed hemothorax. There was a trend toward increased risk of hemothorax in patients with sarcoidosis (*p* = 0.13) and in re-transplantation (*p* = 0.17), compared to the other lung disease groups.

#### Morbidity and mortality

Patients who developed hemothorax experienced worsened morbidity (Table 3). Of the patients who developed hemothorax, 65 % required re-exploration in the operating room, 76 % became hemodynamically unstable and 53 % required vasopressors.

**Table 3:** Morbidity and mortality in patients who developed hemothorax or not

Characteristics	HTX (*N* = 17)	No HTX (*N* = 115)	*P*
Ventilator free days	17	27	0.006
Chest tube LOS (d)	14	10	0.028
ICU LOS (d)	27	8	0.01
Hospital LOS (d)	38	17	0.005

hemothorax (HTX) compared to those who did not develop

hemothorax (No HTX) and the corresponding *p* values

*D* days, *LOS* length of stay

Ventilator-free days, defined as days alive and free from mechanical ventilation, was significantly shorter in the hemothorax group (17 days) compared to the non-hemothorax group (27 days, *p* = 0.006). The patients who developed hemothorax had a longer chest tube requirement from time of transplant of 14 days compared to 10 days in patients who did not develop hemothorax (*p* = 0.028). Patients who developed hemothorax had a significantly increased ICU length of stay (27 days vs. 8 days, respectively, *p* = 0.010) and hospital length of stay (38 days vs. 17 days, respectively, *p* = 0.005) compared to those who did not.

**Fig. 1:**
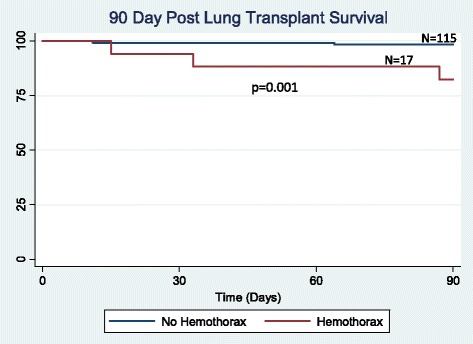
90-day survival after lung transplantation for patients with and without hemothorax

**Fig. 2:**
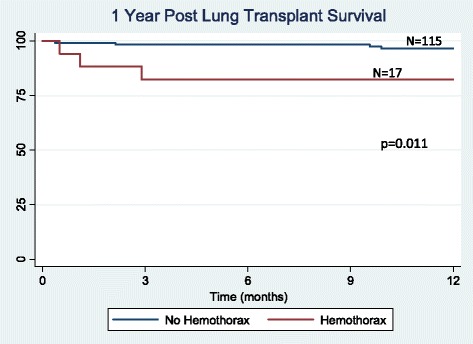
One year survival after lung transplantation for patients with and without hemothorax

**Fig. 3:**
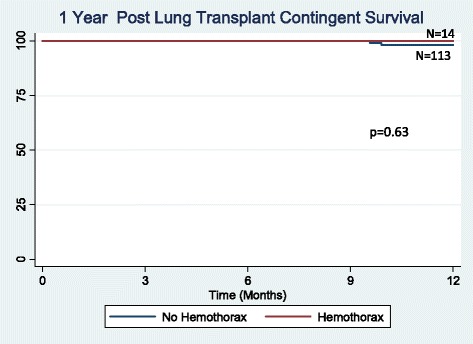
One year survival after lung transplantation for patients with and without hemothorax conditional on survival to 90 days

**Fig. 4:**
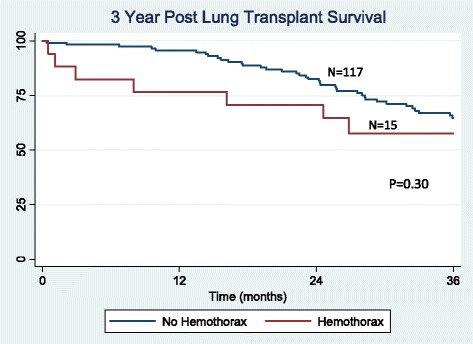
Three year survival after lung transplantation for patients with and without hemothorax

**Fig. 5:**
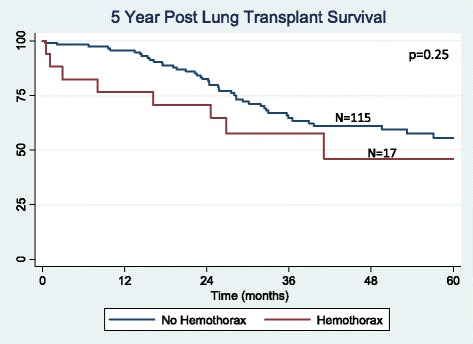
Five year survival after lung transplantation for patients with and without hemothorax

Effects of hemothorax on early, short-term and long-term survival were analyzed. Two patients died as a direct consequence of their hemothorax. Ninety-day survival was significantly reduced in patients developing hemothorax (*p* = 0.001, Fig. 1). Overall 1-year survival was also reduced (*p* = 0.011, Fig. 2). For patients who survived beyond 90 days post-transplant, survival at 1 year was not significantly different between those with and without hemothorax (*p* = 0.63, Fig. 3). There was no significant difference in 3 (*p* = 0.30, Fig. 4) or 5-year survival (*p* = 0.25, Fig. 5). Hemothorax did not influence the overall survival of our lung transplant cohort using Cox proportional hazards modeling, even after adjusting for confounding variables of type of transplant, underlying disease, and age (Table 4).

**Table 4:** Unadjusted and adjusted hazard ratios for death among lung transplant patients

	Hazard Ratio (95 % CI)	*P*
Unadjusted: (*N* = 132)		
Hemothorax	1.55 (0.73–3.28)	0.26
Adjusted*: (*N* = 132)		
Hemothorax	1.69 (0.77–3.70)	0.19
Age	1.01 (0.98–1.04)	0.46
Gender	0.94 (0.54–1.63)	0.81
Type of Transplant	1.04 (0.85–1.27)	0.72
Underlying Disease	0.78 (0.38–1.59)	0.50

*Based on Cox proportional hazards model adjusted for hemothorax, age, gender, type of transplant, and underlying disease

## Discussion

In this study we evaluated the incidence and downstream consequences of significant hemothorax in a heterogeneous population of lung transplant recipients. We provide an in-depth analysis of risk factors for hemothorax, as well as describe the incidence and impact of this complication on both morbidity and survival.

The primary finding of our retrospective review is that post-operative hemothorax adversely affects early survival in lung transplant recipients. With regards to survival, a prior study by Ferrer, et al. published in 2003 analyzed acute and chronic pleural complications in 100 lung transplant recipients and found that the development of hemothorax was associated with increased mortality in a multivariate analysis [5]. The prior study did not perform a rigorous evaluation of the many variables that may be associated with development of hemothorax, which we attempted to do. Another study by Ferrer and colleagues focused specifically on causes of death within 30 days of transplant in patients with COPD [6]. This study found that 30 day mortality was higher in patients with post-operative hemothorax, but did not evaluate the longer-term impact of hemothorax on survival. Our survival analysis using Kaplan-Meier methodology allows an alternative means of looking at the effects of hemothorax on post-transplant survival.

It is unclear if the reduced 90 day survival of the hemothorax group is due directly to the hemodynamic consequences of the hemothorax or the downstream sequelae of a more complex post-operative clinical course. In fact, only two patients experienced cardiac arrest as a direct result of hemothorax. Clearly, multiple complications can arise as an indirect consequence of hemothorax related to surgical re-exploration, longer time on the ventilator, and longer hospital LOS, which may all negatively impact survival in the short term. However, the impact of these parameters on longer term survival appears to dissipate over time. This may explain why 1, 3 and 5-year survival curves in our hemothorax patient population are similar to those without hemothorax.

In addition to the effects on early survival, development of hemothorax had a major impact of morbidity. Time on the ventilator and length of stay, both in the ICU and hospital, were significantly increased. Although not specifically studied, it is likely given these findings that the development of a hemothorax also increases the cost of hospitalization.

Our study also adds to the existing medical literature regarding the incidence of hemothorax post-lung transplant. The incidence of hemothorax post-transplantation has not previously been well-reported and varies significantly amongst studies. Significant hemothorax occurred in 12.9 % of patients in our study. This is markedly different from a review of pleural complications published by Herridge and colleagues in 1995, who reported an incidence of only 1.4 % (1 subpleural hematoma and 1 hemothorax in 138 patients) [7]. Hemothorax complicated the post-transplant course 15 % of the time in the previously mentioned study by Ferrer et al. [5] and 17.9 % of the time in the study by Navarro and colleagues [2]. It is not surprising that the reported rate of hemothorax varies among studies given differences in the definition of hemothorax and populations studied.

It is somewhat surprising that the only particular patient or operative characteristic associated with the development of hemothorax was male gender. It is unclear if this represents a type II error or if male gender somehow predisposes patients to the development of hemothorax. Many characteristics considered traditional risk factors were not found to be significantly associated with the development of hemothorax in our cohort. For instance, the use of cardiopulmonary bypass has been demonstrated to increase the need for intra-operative blood products and to be associated with an increased risk for post-operative bleeding, yet we failed to show an increased likelihood of hemothorax in patients transplanted on cardiopulmonary bypass [3, 8]. Certain disease states, including sarcoidosis and re-transplantation, are classically associated with more difficult surgical dissections, which intuitively would seem to enhance the risk for post-operative hemothorax. Our study does show a trend toward increased risk of hemothorax in these populations, but this failed to reach statistical significance likely due to small numbers in these groups. Indeed, this likely represents a type 1 error due to our relatively small sample size. Thus, further study in larger patient cohorts will help to further inform the literature.

**Fig. 6:**
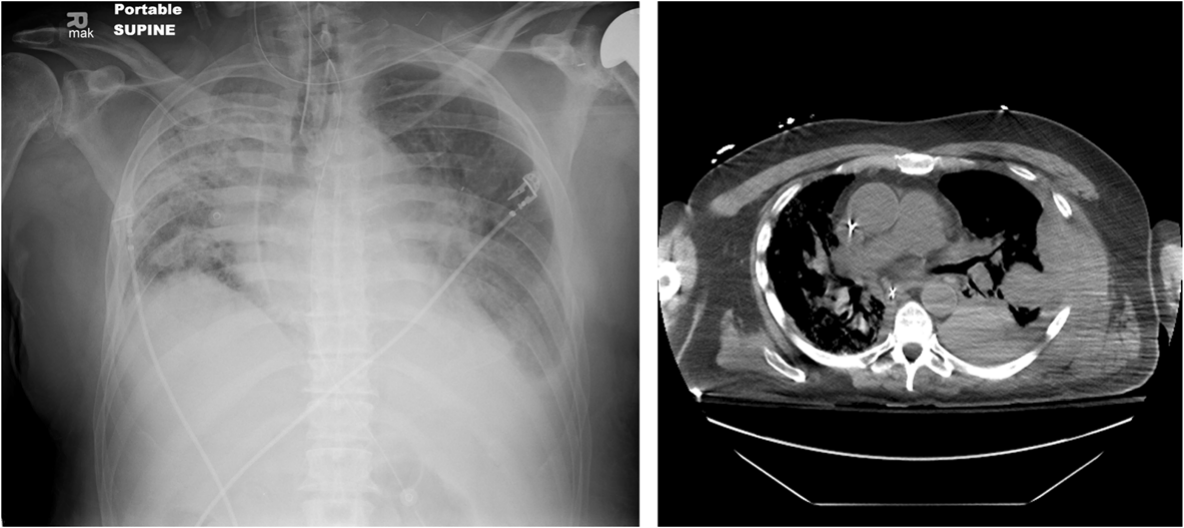
Chest radiograph and CT scan of a patient with left hemothorax resulting in hemodynamic instability

To our knowledge, our group is the first to address the timing of the development of hemothorax post-transplant. Hemothorax is most commonly thought of as a post-surgical complication that develops in the immediate peri-operative period. Somewhat surprisingly, 70.5 % of the patients in our study who developed hemothorax, did so greater than 48 h post-operatively. In fact, the median time to the development of hemothorax post-transplant was 8 days. The majority of patients with “delayed” hemothorax (83.3 %) were on some form of anticoagulation. Given that hemothorax may present in a delayed fashion, at a time when transplant recipients are at risk for other post-operative complications including nosocomial infection, acute rejection, and venous thromboembolism, clinicians must maintain a high index of suspicion for this to ensure timely intervention. Anecdotally, even sizeable, clinically significant hemothoraces may be difficult to appreciate on chest radiography (Fig. 6). Clinicians should have a low threshold to obtain a chest CT in transplant recipients with any hemodynamic instability, particularly in conjunction with unexplained drops in hemoglobin/hematocrit and/or dense pleural-based infiltrates.

Our study has several limitations. All patient data reviewed is from a single center which may limit generalizability. In particular, surgical techniques and management of anticoagulants may vary amongst centers and could impact the incidence of hemothorax encountered. Additionally, our center performs a higher proportion of transplants for ILD than most centers which may influence generalizability. Our data set includes very few patients managed with ECMO support. Additional studies will be required in this specific population to determine the effects on bleeding risk. The sample size is also relatively small, although on par with prior studies. It is likely that we didn’t capture all hemothoraxes, specifically those that did not require an intervention. While it is generally expected that there might be some bleeding post-operatively, how much blood and at what point should this be regarded as a post-operative hemothorax is difficult to define. We perhaps set the bar a little high in our definition of “significant hemothorax” and therefore cannot attest to the incidence or impact of lesser hemothoraxes. Definitions of “clinically significant” hemothorax may vary among studies. We feel that the definition we chose has real world applicability and is thus an appropriate definition which may be used as a standard for future similar studies. Finally, the study is retrospective in nature and it is possible that cases of significant hemothorax were missed. Given the definition used and the rigorous chart review performed, we feel it is unlikely that cases meeting the definition were not detected.

## Conclusions

In conclusion, a high index of suspicion for hemothorax should be maintained post-transplant as it negatively affects patient outcomes, particularly in the subsequent weeks to months. Hemothorax may develop in a delayed manner, and therefore clinicians must remain vigilant outside the immediate post-operative window, particularly in patients on anticoagulation. Future studies with larger sample sizes are needed to further elucidate and abrogate risk factors for hemothorax. Equally important are studies to guide patient specific risk stratification for post-operative anticoagulation and optimize strategies to best strike the tenuous balance of need for anticoagulation and risk of bleeding.

## Abbreviations

COPD: Chronic obstructive pulmonary disease
DVT: Deep vein thrombosis
ICU: Intensive care unit
ILD: Interstitial lung disease
INR: Intentional normalized ratio
IPF: Idiopathic pulmonary fibrosis
LAM: Lymphangioleiomyomatosis
LAS: Lung allocation system
LOS: Length of stay
mPAP: Mean pulmonary artery pressure
P/F ratio: Pulmonary arterial oxygen saturation to fraction of inspired oxygen ratio
PTT: Partial thromboplastin time
VATS: Video-assisted thorascopic surgery
